# The Host NADase CD38 Promotes JEV Replication by Targeting the NAD+/SIRT1 Axis

**DOI:** 10.3390/microorganisms14040796

**Published:** 2026-04-01

**Authors:** Yuanyuan Yang, Ruiqin Zhang, Xinran Li, Xinlei Liu, Yu Dai, Yu Gu, Jiahui Li, Haodong Chen, Yi Zheng, Rui Wu

**Affiliations:** 1 Research Center for Swine Diseases, College of Veterinary Medicine, Sichuan Agricultural University, Chengdu 611130, China; 15111958566@163.com (Y.Y.); zhangruiqin2026@163.com (R.Z.); 2023203030@stu.sicau.edu.cn (X.L.); leslieliu1998@163.com (X.L.); daiyu0102@163.com (Y.D.); gu15198666942@163.com (Y.G.); hui007ljh@163.com (J.L.); 18113013960@163.com (H.C.); zhengyi132@126.com (Y.Z.); 2 Sichuan Science-Observation Experiment Station of Veterinary Drugs and Veterinary Diagnostic Technology, Ministry of Agriculture, Chengdu 611330, China; 3 National Animal Experiments Teaching Demonstration Center, Sichuan Agricultural University, Chengdu 611330, China

**Keywords:** Japanese encephalitis virus, CD38, NAD+ metabolism, SIRT1/p53 axis

## Abstract

The manipulation of host cellular metabolism is a key strategy for flaviviruses like Japanese encephalitis virus (JEV) to establish a productive infection. This study identifies the host NADase CD38 as a central regulator of this process. Using a CRISPR/Cas9-generated CD38 knockout (KO) TM3 cell model, we found that CD38 deficiency significantly restricted the production of infectious viral particles. While loss of CD38 also partially impaired viral entry, our central finding is that CD38 primarily promotes JEV infection by suppressing a host-intrinsic metabolic defense. We show that CD38 deficiency leads to a surge in intracellular NAD+, which sustains SIRT1 activity and inactivates p53, thereby blocking the mitochondrial apoptosis required for viral propagation. The dominance of this metabolic axis was confirmed through bidirectional pharmacological interventions; while SIRT1 inhibition using EX527 restored JEV replication, SIRT1 activation using SRT1720 suppressed it in wild-type cells. Our work reveals that JEV hijacks the CD38-NAD+-SIRT1-p53 axis to overcome host metabolic defenses in reproductive cell models, establishing CD38 as a promising therapeutic target.

## 1. Introduction

Japanese encephalitis virus (JEV) is a mosquito-borne flavivirus that causes acute viral encephalitis in humans and reproductive failure in swine [1]. Historically, since the first confirmed case in Japan (1924), JEV has driven large-scale epidemics across Asia—including South Korea (1933), China (1940), the Philippines (1950), and India (1955)—establishing itself as a persistent public health threat [2,3]. To sustain such widespread pathogenicity and efficient replication, JEV actively modulates host cellular pathways, triggering severe stress responses such as oxidative stress, endoplasmic reticulum (ER) stress, and apoptosis [4,5,6]. While often a host defense, flaviviruses can exploit apoptotic signaling to facilitate viral particle release (egress) [7]. Thus, metabolic checkpoints controlling apoptosis are critical for viral propagation. These stress responses disrupt cellular homeostasis and impose a substantial metabolic demand on the host. For example, the Zika virus has been shown to manipulate host NAD+ bioenergetics to evade immune defenses [8]. Consequently, enzymes regulating NAD+, particularly CD38, may play a critical role in infection outcome.

CD38 is a multifunctional transmembrane glycoprotein and a key regulator of nicotinamide adenine dinucleotide (NAD+) metabolism [9,10]. As one of the primary NAD+-consuming enzymes in mammals, CD38 competes with other NAD+-dependent enzymes, such as Sirtuins (SIRTs) and PARPs, for the limited intracellular NAD+ pool [11,12]. Consequently, CD38 limits NAD+ bioavailability, thereby modulating the function of downstream sensors like SIRT1 [13,14].

Nicotinamide adenine dinucleotide (NAD+) is a central coenzyme in cellular redox reactions and serves as a critical signaling molecule for a range of cellular processes, including DNA repair, inflammation, and cell survival [15,16]. The intracellular NAD+ pool is tightly regulated, with its availability acting as a rheostat for NAD+-dependent enzymes. Among these, the sirtuin family of deacetylases, particularly SIRT1, functions as a key sensor of cellular NAD+ levels [17].

SIRT1, an NAD+-dependent class III histone deacetylase, plays a pivotal role in cellular survival by deacetylating key substrates, including the tumor suppressor p53 [18]. Deacetylation of p53 by SIRT1 inhibits its transcriptional activity, thereby suppressing p53-mediated apoptosis and promoting cell survival [19]. Previous studies have established a competitive axis between CD38 and SIRT1: high CD38 expression depletes cellular NAD+, limiting SIRT1 activity and enhancing p53 acetylation [20]. Conversely, CD38 deficiency or inhibition restores NAD+ levels, reactivating SIRT1 and suppressing p53-dependent signaling [21]. However, whether JEV hijacks the CD38-NAD+-SIRT1-p53 axis to facilitate its replication remains unexplored.

In this study, given the clinical relevance of JEV in porcine reproductive pathogenesis, we utilized TM3 Leydig cells as a model [22]. We aimed to investigate the role of CD38 in JEV infection. We demonstrate that JEV hijacks CD38 to deplete NAD+, thereby inactivating the SIRT1 defense mechanism and promoting viral replication via p53-dependent apoptosis.

## 2. Materials and Methods

### 2.1. Cells and Viruses

TM3 Leydig cells, BHK-21 cells, and HEK-293T cells were kindly provided by the Swine Disease Research Center, Sichuan Agricultural University (Chengdu, China). Cells were cultured in DMEM supplemented with 10% fetal bovine serum (FBS, ExCell Bio, Suzhou, China) and 1% penicillin-streptomycin (Beyotime, Shanghai, China) at 37 °C with 5% CO_2_. The JEV strain SCYA201201-1 (GenBank: KU508408.1) was propagated in BHK-21 cells and titrated by plaque assay.

### 2.2. Generation of CD38-Knockout Cells

CD38-knockout (KO) TM3 cells were generated using the CRISPR/Cas9 system. Specific sgRNAs targeting the first exon of the *CD38* gene (sgRNA-F: 5′-TAGGGATCGTGGTCATACTTCTG-3′) were cloned into the LentiCRISPR-V2 plasmid. Lentiviral particles were produced in HEK-293T cells, and TM3 cells were infected and selected with puromycin. Knockout efficiency was verified by DNA sequencing and Western blotting.

psPAX (12260), pMD2.G (12259), and LentiCRISPR-V2 (52961) were purchased from Addgene (Cambridge, MA, USA).

### 2.3. Western Blot Analysis

Cells were lysed in RIPA buffer containing PMSF. Protein concentration was determined using a BCA kit (Thermo Fisher Scientific, Shanghai, China). Lysates were separated by SDS-PAGE and transferred to PVDF membranes. Membranes were blocked with 5% skim milk and incubated overnight at 4 °C with primary antibodies. After incubation with HRP-conjugated secondary antibodies, bands were visualized using an ECL detection system. β-actin served as the loading control.

Anti-E (HL2517) from Gene Tex (San Antonio, TX, USA); Anti-β-actin (AC026), HRP-conjugated goat anti-rabbit (AS014), and HRP-conjugated goat anti-mouse (AS003) from Abclonal (Wuhan, China); Anti-BAX (50599-2-Ig), and anti-P53 (60283-2-Ig) from Proteintech (Wuhan, China); Anti-acetyl-P53 (YM8727) from Immunoway Biotechnology (Plano, TX, USA); anti-caspase-3 (ab32150) from Abcam (Cambridge, MA, USA); Anti-SIRT1 (04212) from UpingBio (Hangzhou, China); anti-BCL-2 (HA721235) from Huabio (Hangzhou, China).

### 2.4. RNA Extraction and qRT-PCR

Total RNA was extracted using the Trizol reagent (Sangon Biotech, Shanghai, China). cDNA synthesis was performed using the PrimeScript™ RT Reagent Kit (Takara Bio, Otsu, Shiga, Japan). Quantitative real-time PCR (qRT-PCR) was conducted using TB Green Premix Ex Taq™ II (Takara, Otsu, Japan) on a LightCycler 96 System (Roche, Basel, Switzerland). Relative gene expression was calculated using the 2^−ΔΔCt^ method normalized to GAPDH. Primer sequences are listed in Table 1.

### 2.5. Viral Attachment and Internalization Assay

For attachment assays, cells were incubated with JEV (MOI = 10) at 4 °C for 2 h. Unbound virions were removed by washing with cold PBS, and cell-associated viral RNA was quantified by qRT-PCR. For internalization assays, cells were shifted to 37 °C for 2 h after binding. Non-internalized virus was removed by washing with acidic citrate buffer (pH 3.0), and internalized viral RNA was quantified.

### 2.6. TCID_50_ Assay

To determine infectious viral titers, supernatants from infected cells were collected at the indicated time points. BHK-21 cells were seeded in 96-well plates and infected with 10-fold serial dilutions of the viral supernatants. After 4–5 days, the cytopathic effect (CPE) was observed under a microscope, and the 50% tissue culture infectious dose (TCID_50_) was calculated using the Reed-Muench method.

### 2.7. Immunofluorescence Assay (IFA)

Cells seeded on coverslips were infected with JEV (MOI = 1) for 24 h. Cells were fixed with 4% paraformaldehyde, permeabilized with 0.1% Triton X-100 (Beyotime, Shanghai, China), and blocked with 2% BSA (Beyotime, Shanghai, China). Samples were incubated with anti-JEV E antibody followed by FITC-conjugated secondary antibody. Nuclei were stained with DAPI. Images were captured using an Olympus BX63 fluorescence microscope (Tokyo, Japan).

### 2.8. Cell Proliferation Assay

Cell proliferation was assessed using the BeyoClick™ EdU Cell Proliferation Kit (Beyotime, Shanghai, China) according to the manufacturer’s instructions. EdU-positive cells were visualized by fluorescence microscopy and quantified using ImageJ software (Version 1.53e).

### 2.9. NAD+ Detection

Intracellular NAD+ concentration was determined using an NAD+/NADH Assay Kit with WST-8 (S0175, Beyotime) according to the manufacturer’s protocol. Briefly, cell lysates were prepared, and absorbance was measured at 450 nm using a microplate reader. NAD+ levels were calculated by subtracting NADH from the total NAD+/NADH pool and normalized to protein concentration.

### 2.10. Statistical Analysis

All data are presented as the means ± standard deviation (SD) from three independent biological replicates (*n* = 3) for each experiment. Statistical analyses were performed using GraphPad Prism version 5.0 (GraphPad Software, San Diego, CA, USA). The selection of statistical tests was based on the specific experimental design. For comparisons between two independent groups, an unpaired, two-tailed Student’s *t*-test was used. For comparisons among three or more groups with a single independent variable, such as cell viability assays, a one-way analysis of variance (ANOVA) followed by Tukey’s post hoc test was employed. Furthermore, for data involving two independent variables, including viral RNA growth kinetics and TCID_50_ over time, a two-way ANOVA followed by Bonferroni’s multiple comparisons test was utilized. A *p*-value of less than 0.05 was considered statistically significant (* *p* < 0.05, ** *p* < 0.01, *** *p* < 0.001, ns = not significant).

## 3. Results

### 3.1. Generation and Characterization of CD38-Knockout Cells

**Figure 1:**
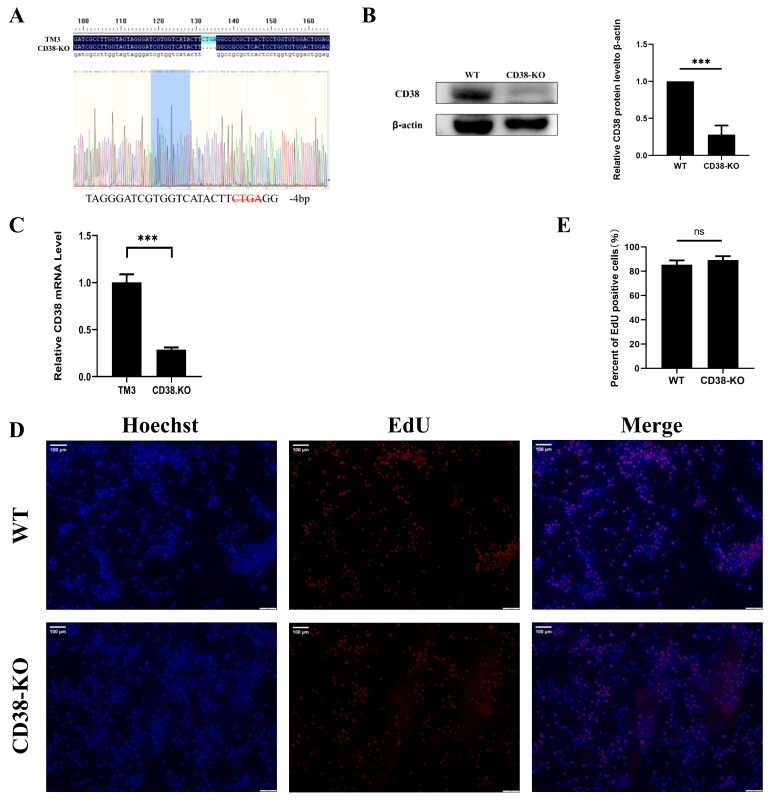
Generation and characterization of CD38-knockout cells. (**A**) Sanger sequencing validation of CD38 knockout in wild-type (WT) and CD38-KO cells. The blue-highlighted sequence marks the sgRNA target region; the dotted line and red-labeled bases indicate the 4 bp deletion in CD38-KO cells (−4 bp). The sequencing peak map uses standard color coding: adenine (A, green), thymine (T, red), cytosine (C, blue), guanine (G, black).(**B**) Detection of CD38 protein knockout efficiency by Western blot analysis. (**C**) CD38 mRNA levels were measured by RT-qPCR and normalized to WT cells. (**D**) WT and CD38-KO cells were incubated for 2 h after EdU loading. After the click reaction, pictures were taken using a fluorescence microscope. 5-Ethynyl-2′-deoxyuridine (EdU)-positive cells were shown in red, and the nuclei were shown in blue. (**E**) At least three images were captured for each sample to calculate the percentage of EdU-positive cells, with the average reported as the final data. Data represent the means ± SD from three independent biological replicates (*n* = 3). *** *p* < 0.001. ns, not significant.

A CD38 knockout (KO) cell line was established using the CRISPR/Cas9 system and designated as CD38-KO cells. Genomic DNA extracted from both WT (Wild type) and CD38-KO cells was sequenced. Sequencing of the target locus in CD38-KO cells revealed a 4 bp deletion, confirming a frameshift mutation (Figure 1A), confirming successful Cas9-mediated cleavage and the introduction of a frameshift mutation. To verify the knockout at both protein and transcriptional levels, Western blotting and RT-qPCR were performed. As shown in Figure 1B,C, CD38 protein was undetectable, and its mRNA level was significantly reduced in CD38-KO cells compared to WT cells, demonstrating successful CD38 knockout. To rule out proliferation defects, we conducted EdU incorporation assays. Newly synthesized DNA labeled with Alexa Fluor 555 showed red fluorescence, while nuclei were counterstained with Hoechst 33342 (blue). Comparison between CD38-KO and WT cells indicated that CD38 knockout did not significantly affect cellular proliferation (Figure 1D,E).

### 3.2. CD38 Deficiency Restricts JEV Replication and Cytopathic Effects

**Figure 2:**
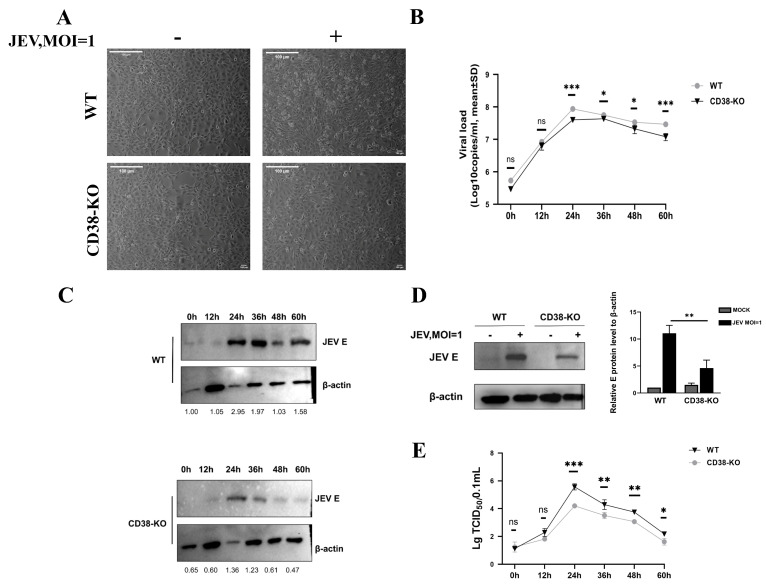
CD38 deficiency restricts JEV replication and cytopathic effects. (**A**) Observation of cell status changes 24 h after JEV infection (MOI = 1). (**B**) Supernatants from infected TM3 and CD38-KO cells were collected at the indicated hpi; RT–qPCR of the E gene was performed to quantify viral load, and growth curves were generated. (**C**) WT and CD38-KO cells were infected with JEV (MOI = 1) at different times, and E was analyzed at the protein level by Western blot analysis. (**D**) TM3 and CD38-KO cells were infected with JEV (MOI = 1) for 24 h; E protein levels were analyzed by Western blot analysis. (**E**) Supernatants from JEV-infected WT and CD38-KO TM3 cells (MOI = 1) were collected at 0, 12, 24, 36, 48, and 60 h post-infection. Infectious viral titers were determined by a standard TCID_50_ assay on BHK-21 cells. Data represent the means ± SD from three independent biological replicates (*n* = 3). * *p* < 0.05, ** *p* < 0.01, *** *p* < 0.001; ns, not significant.

To evaluate the replication kinetics of JEV, both wild-type (WT) TM3 and CD38-KO cells were infected with JEV at an MOI of 1 and monitored for cytopathic effects. JEV infection (MOI = 1) induced severe cytopathic effects (CPE), including cell shrinkage and lysis, in wild-type (WT) TM3 cells by 24 h post-infection. In contrast, CD38-KO cells showed significantly attenuated CPE (Figure 2A). Consistently, CD38 deficiency severely restricted viral proliferation, with viral RNA loads remaining significantly lower in KO cells compared to WT counterparts throughout the infection course (Figure 2B). Western blot analysis corroborated this, showing a marked reduction in viral E protein accumulation (Figure 2C,D). Because viral RNA levels do not always directly reflect the production of viable virions, we further assessed the release of infectious viral particles using a TCID_50_ assay. In perfect agreement with the RT-qPCR data, the infectious viral titers in the supernatants of CD38-KO cells were significantly reduced at 24, 36, 48, and 60 h post-infection compared to those from WT cells (Figure 2E). Taken together, these results demonstrate that CD38 deficiency potently restricts JEV replication and the production of infectious progeny.

### 3.3. CD38 Deficiency Impairs JEV Attachment and Internalization

**Figure 3:**
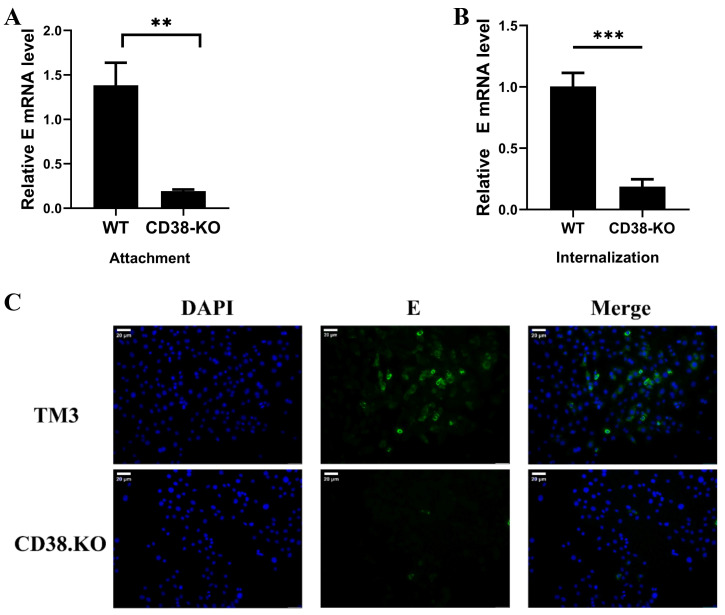
CD38 deficiency impairs JEV attachment and internalization. (**A**) TM3 and CD38-KO cells were inoculated with JEV (MOI = 10) at 4 °C for 2 h; excess viruses were removed by washing with cold PBS. (**B**) Viruses were bound to cells as described above and then shifted to 37 °C for 2 h; noninternalized viruses were removed by washing cells with citrate buffer (pH = 3). RT-qPCR of the E gene was used to determine the mRNA levels of the virus. (**C**) IFA results of binding and internalization processes after the cells were inoculated with JEV (MOI = 10). Scale bar = 20 μm. Data represent the means ± SD from three independent biological replicates (*n* = 3). ** *p* < 0.01, *** *p* < 0.001.

To determine whether this restriction occurred at the entry stage, we performed attachment and internalization assays. CD38-KO cells showed a marked defect in both viral adsorption at 4 °C (Figure 3A) and internalization at 37 °C (Figure 3B), consistent with immunofluorescence observations (Figure 3C). While CD38 facilitates attachment, we next investigated whether CD38 regulates post-entry replication via metabolic reprogramming.

### 3.4. CD38 Knockout Prevents JEV-Induced NAD+ Depletion and Activates the SIRT1/p53 Signaling Axis

**Figure 4:**
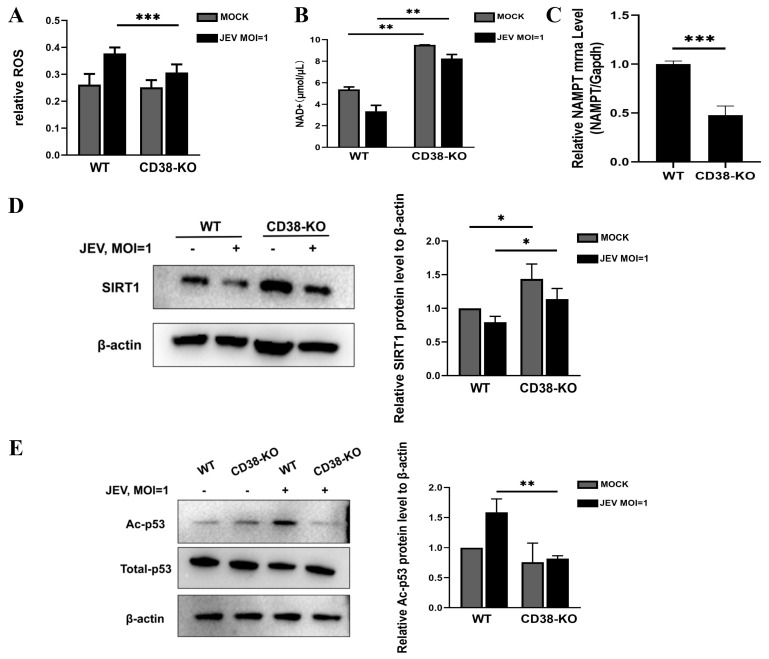
CD38 knockout prevents JEV-induced NAD+ depletion and activates the SIRT1/p53 signaling axis. (**A**) Intracellular ROS levels detected by DCFH-DA staining (Quantification). (**B**) Intracellular NAD+ levels. (**C**) Relative mRNA levels of NAMPT were determined by RT-qPCR and normalized to GAPDH. (**D**) Western blot analysis of SIRT1. (**E**) Western blot analysis of acetylated p53 (K382) and total p53. Data represent the means ± SD from three independent biological replicates (*n* = 3). * *p* < 0.05, ** *p* < 0.01, *** *p* < 0.001.

Given the role of CD38 in metabolism, we investigated intracellular signaling pathways. JEV infection triggers severe oxidative stress and metabolic imbalance. We found that while JEV induced robust reactive oxygen species (ROS) accumulation in WT cells, this response was significantly blunted in CD38-KO cells (Figure 4A). Mechanistically, CD38 deficiency led to the accumulation of intracellular NAD+, preventing its depletion by JEV (Figure 4B). To further investigate the dynamics of the NAD+ metabolic network, we assessed the mRNA levels of NAMPT, the rate-limiting enzyme in the NAD+ salvage pathway. We observed that NAMPT transcription was significantly down-regulated in CD38-KO cells compared to WT cells (Figure 4C), suggesting a compensatory negative feedback loop in response to the massive intracellular NAD+ accumulation. This high NAD+ state sustained SIRT1 expression (Figure 4D). Consistent with SIRT1 activation, p53 acetylation (K382) was abolished in CD38-KO cells (Figure 4E). These results suggest that CD38 deficiency creates an anti-oxidant and anti-apoptotic metabolic environment.

### 3.5. Bidirectional Modulation of SIRT1 Dictates JEV Replication via the Mitochondrial Apoptotic Pathway

**Figure 5:**
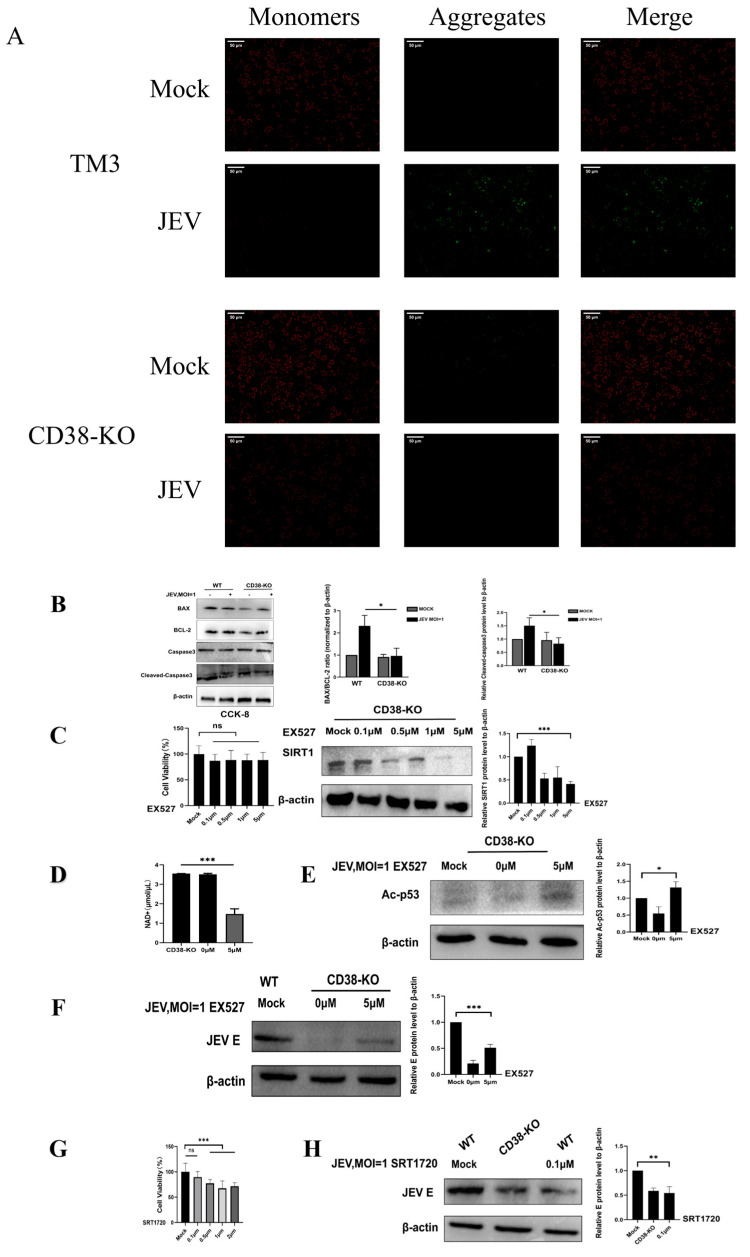
Bidirectional modulation of SIRT1 dictates JEV replication via the mitochondrial apoptotic pathway (**A**) Mitochondrial membrane potential (ΔΨm) detected by JC-1 staining. Scale bar = 50 μm. (**B**) Western blot analysis of apoptosis-related markers (Bax, Bcl-2, and Cleaved Caspase-3). (**C**) Effect of EX527 on cell viability (CCK-8, left) and SIRT1 protein levels (Western blot, right). (**D**) NAD+ levels after EX527 treatment (5 μM). (**E**) Western blot of p53 acetylation recovery. (**F**) Rescue of JEV E protein expression by EX527. (**G**) CCK-8 assay evaluating SRT1720 cytotoxicity in WT cells. (**H**) SRT1720 (0.1 μM) suppresses JEV E protein expression in WT cells, with CD38-KO cells as a restriction control. Data are means ± SD from three independent biological replicates (*n* = 3). * *p* < 0.05, ** *p* < 0.01, *** *p* < 0.001; ns, not significant.

To link the metabolic axis to viral replication, we assessed the mitochondrial apoptotic pathway. CD38-KO cells maintained mitochondrial membrane potential (ΔΨm) (Figure 5A) and showed reduced activation of the apoptotic cascade, characterized by a suppressed Bax/Bcl-2 ratio and inhibited Caspase-3 cleavage (Figure 5B). To validate the metabolic mechanism, we employed the SIRT1 inhibitor EX527. We first confirmed that EX527 (up to 5 μM) showed no cytotoxicity and effectively modulated SIRT1 activity (Figure 5C). Treatment with 5 μM EX527 restored viral replication and phenotype. It depleted the accumulated NAD+ (Figure 5D), restored p53 acetylation (Figure 5E). Notably, EX527 treatment restored p53 acetylation and JEV E protein expression (Figure 5F). Importantly, this rescue occurred despite the entry defect in CD38-KO cells, suggesting that the NAD+/SIRT1 metabolic barrier is the dominant restriction factor post-entry. Conversely, we tested whether stimulating SIRT1 could restrict JEV in WT cells. Using the SIRT1 activator SRT1720 at a pre-determined non-toxic concentration (0.1 μM, Figure 5G), we observed a partial suppression of JEV E protein expression, which effectively phenocopied the CD38-KO antiviral state (Figure 5H). The ability to toggle JEV replication via SIRT1 inhibition and activation provides compelling evidence that the NAD+/SIRT1 metabolic barrier is the dominant post-entry restriction factor.

## 4. Discussion

While JEV is primarily neurotropic, it causes significant reproductive failure in swine, leading to orchitis and viral shedding in semen. TM3 Leydig cells are a well-established model for studying JEV-induced reproductive pathogenesis [22]. Utilizing this model, we identified the host ectoenzyme CD38 as a critical proviral factor for JEV. Beyond its partial role in viral entry, we found that CD38 primarily serves as a metabolic checkpoint exploited by JEV. Flaviviruses frequently target host bioenergetics; for instance, Zika virus pathogenesis is closely linked to altered NAD+ pools [8]. Our results extend this concept, demonstrating that JEV specifically relies on CD38-mediated NAD+ consumption to dismantle intrinsic host defenses.

The life cycle of JEV begins with receptor recognition and entry [23]. Our study found that CD38 knockout significantly inhibits the adsorption and internalization of JEV. Previous studies have shown that JEV entry relies on specific host factors, such as CD4 [24]. While CD38 is not a known receptor for JEV, its high expression on the cell surface may facilitate the clustering of entry complexes or modulate the membrane microenvironment [25]. Our data suggest that CD38 deficiency creates a non-permissive physical environment for JEV adsorption, serving as the first line of defense. However, given the complexity of viral replication, the precise molecular interaction between CD38 and JEV entry receptors warrants further investigation.

We demonstrated that CD38 knockout prevents JEV-induced NAD+ depletion, resulting in a robust intracellular NAD+ accumulation. Interestingly, this surge in NAD+ was accompanied by a significant down-regulation of NAMPT transcription. As NAMPT is the rate-limiting enzyme in the NAD+ salvage pathway, its suppression likely reflects a compensatory negative feedback loop triggered by the loss of CD38. This coordinated remodeling of the NAD+ flux suggests that the host cell actively attempts to restore metabolic homeostasis when a major NAD+ consumer is ablated [26]. However, this high-NAD+ state inadvertently creates a highly non-permissive environment for JEV. This high-NAD+ state directly sustains SIRT1 activity, which subsequently deacetylates and inactivates the pro-apoptotic factor p53 at lysine 382. Flaviviruses frequently exploit host apoptotic signaling to facilitate the egress and dissemination of infectious viral particles [27,28,29]. In wild-type infection, JEV-induced NAD+ depletion inhibits SIRT1, driving p53 hyperacetylation and triggering the mitochondrial apoptotic cascade (Bax/Bcl-2 and Caspase-3). By preserving SIRT1 function, CD38 deficiency maintains p53 in an unacetylated state, effectively neutralizing this virus-induced apoptosis and drastically reducing the release of infectious progeny, as evidenced by our TCID_50_ data.

Crucially, our bidirectional pharmacological interventions confirmed the absolute dominance of this metabolic axis. Inhibiting SIRT1 with EX527 in CD38-KO cells reversed the antiviral state and restored JEV replication, whereas stimulating SIRT1 with SRT1720 in wild-type cells successfully phenocopied the CD38-KO restriction. This uncoupling of the metabolic defense from the physical entry defect is a significant finding. It demonstrates that intracellular metabolic reprogramming serves as an independent and highly potent restriction mechanism. Furthermore, the robust antiviral effect of SRT1720 underscores the therapeutic potential of SIRT1 activators, which have shown promise against other viral infections by bolstering host-intrinsic defenses [30].

We acknowledge certain limitations in our current experimental model. First, because JEV pathogenesis in humans is predominantly driven by central nervous system infection, the metabolic baselines of Leydig cells may differ from those of neuronal tissues. Neurons have uniquely high bioenergetic demands and distinct NAD+ pool compartmentalization [31]. Determining whether the CD38-NAD+-SIRT1-p53 axis plays an equally restrictive role in JEV-infected neurons is a critical avenue for future in vivo studies. Second, NAD+ metabolism is a highly complex network. While we identified the interplay between CD38, NAMPT, and SIRT1, other crucial regulators, such as PARP family enzymes—which are major NAD+ consumers rapidly activated during virus-induced DNA damage—were not evaluated [32]. Future studies employing targeted metabolomics are needed to fully map the NAD+ landscape during flavivirus infections.

In conclusion, our study establishes CD38 as a key proviral factor that JEV hijacks to facilitate its life cycle. CD38 acts as a metabolic switch to suppress the host’s intrinsic NAD+/SIRT1/p53 antiviral defense axis. These findings deepen our understanding of flavivirus-host metabolic interactions and highlight the CD38-SIRT1 pathway as a promising therapeutic target for host-directed antiviral therapies.

## Figures and Tables

**Table 1:** Primer pairs used for plasmid construction and quantitative real-time RT-PCR.

Primer Name	Forward (5′ → 3′)	Reverse (5′ → 3′)
Q-E	CAGTGGAGCCACTTGGGTG	TTGTGAGCTTCTCCTGTCG
Q-CD38	AAGGAGCTTCCAGTAACGCAT	GATGGGTGCTCAGGGTTCTT
Q-GAPDH	CATCACTGCCACCCAGAAGAC	ATTGGGGGTAGGAACACGGA
Q-NAMPT	CTGTGGCGGGAATTGCTCTA	CCCAAGCCGTTATGGTACTGT

## Data Availability

The original contributions presented in this study are included in the article. Further inquiries can be directed to the corresponding author.
